# Design Considerations for mHealth Programs Targeting Smokers Not Yet Ready to Quit: Results of a Sequential Mixed-Methods Study

**DOI:** 10.2196/mhealth.6845

**Published:** 2017-03-10

**Authors:** Jennifer B McClure, Jaimee Heffner, Sarah Hohl, Predrag Klasnja, Sheryl L Catz

**Affiliations:** ^1^ Kaiser Permanente Washington Health Research Institute (formerly, Group Health Research Institute) Seattle, WA United States; ^2^ Fred Hutchinson Cancer Research Center Seattle, WA United States; ^3^ School of Public Health University of Washington Seattle, WA United States; ^4^ Betty Irene School of Nursing University of California, Davis Sacramento, CA United States

**Keywords:** tobacco, smoking cessation, telemedicine, mobile health, smartphone, motivation

## Abstract

**Background:**

Mobile health (mHealth) smoking cessation programs are typically designed for smokers who are ready to quit smoking. In contrast, most smokers want to quit someday but are not yet ready to quit. If mHealth apps were designed for these smokers, they could potentially encourage and assist more people to quit smoking. No prior studies have specifically examined the design considerations of mHealth apps targeting smokers who are not yet ready to quit.

**Objective:**

To inform the user-centered design of mHealth apps for smokers who were not yet ready to quit by assessing (1) whether these smokers were interested in using mHealth tools to change their smoking behavior; (2) their preferred features, functionality, and content of mHealth programs addressing smoking; and (3) considerations for marketing or distributing these programs to promote their uptake.

**Methods:**

We conducted a sequential exploratory, mixed-methods study. Qualitative interviews (phase 1, n=15) were completed with a demographically diverse group of smokers who were smartphone owners and wanted to quit smoking someday, but not yet. Findings informed a Web-based survey of smokers from across the United States (phase 2, n=116). Data were collected from April to September, 2016.

**Results:**

Findings confirmed that although smokers not yet ready to quit are not actively seeking treatment or using cessation apps, most would be interested in using these programs to help them reduce or change their smoking behavior. Among phase 2 survey respondents, the app features, functions, and content rated most highly were (1) security of personal information; (2) the ability to track smoking, spending, and savings; (3) content that adaptively changes with one’s needs; (4) the ability to request support as needed; (5) the ability to earn and redeem awards for program use; (6) guidance on how to quit smoking; and (7) content specifically addressing management of nicotine withdrawal, stress, depression, and anxiety. Results generally did not vary by stage of change for quitting smoking (precontemplation vs contemplation). The least popular feature was the ability to share progress via social media. Relevant to future marketing or distribution considerations, smokers were price-sensitive and valued empirically validated programs. Program source, expert recommendations, and user ratings were also important considerations.

**Conclusions:**

Smokers who are not yet ready to quit represent an important target group for intervention. Study findings suggest that many of these individuals are receptive to using mHealth tools to reduce or quit smoking, despite not having made a commitment to quit yet. The preferences for specific mHealth intervention features, functionality, and content outlined in this paper can aid addiction treatment experts, design specialists, and software developers interested in creating engaging interventions for smokers who want to quit in the future but are not yet committed to this important health goal.

## Introduction

Cigarette smoking is the leading preventable cause of death and illness in the United States [1] and a significant health issue worldwide [2]. In the United States, an estimated 17% of adults are regular smokers [3]. Most of these people (69%) want to quit smoking someday [4] but they are not yet ready to quit or seek treatment. In fact, most current smokers are characterized as being in the precontemplation or contemplation stages of change, meaning they either are not thinking about quitting or have no interest in quitting in the near term. Typically, only a third or less of current smokers report that they are in the preparation stage of change [5-8], meaning they are planning to quit smoking in the next month.

Whereas most smokers are not ready to quit and are not seeking treatment to quit smoking, most public health smoking interventions and nicotine dependence treatment programs—including smoking cessation apps—are designed for those smokers who are ready to quit in the near term. These programs are typically designed to help people take action but do not necessarily include the support, encouragement, or information smokers need to move from a position of wanting to quit someday to being ready to quit now or to help smokers cut back, but not quit, smoking. As a result, these programs may also have little appeal to smokers who are ambivalent about quitting in the near term. Utilization data are limited but it seems unlikely that smokers who are not actively thinking about quitting smoking are downloading or using cessation apps. For example, in a recent multinational survey of smokers who downloaded a cessation app, 77% were ready to quit in the next month (preparation stage of change) [9].

In contrast, we believe smokers who are not ready to quit in the near term may be receptive to mHealth tools if these tools were better designed to address their needs and interests, particularly among people who typically use mobile devices already. In prior research, we found precontemplative and contemplative smokers were receptive to both counseling [10,11] and Internet-based programs [12] when these programs were designed to help them make informed decisions about their smoking behavior (as opposed to quitting, per se), and as a consequence, many ultimately quit smoking. Thus, we hypothesized that smokers who are not yet ready to quit could also be interested in using mHealth apps, if these programs are designed to address their needs and interests and marketed or distributed in a way to encourage their use when people are not actively seeking treatment.

Increasing attention is being focused on how to design appealing and effective mHealth programs for smokers who are ready to quit and on identifying smokers’ preferred mHealth features [13-18]; however, little is known about the mHealth needs and preferences of smokers who are not yet ready to quit. This insight is critical to designing appealing and effective mHealth-based, public health interventions in the future.

The goal of this research was to inform the user-centered design of mHealth tools for smokers who are not yet ready to quit by assessing (1) whether these smokers are interested in using mHealth apps to change their smoking behavior; (2) their preferred features, functionality, and content for these programs; and (3) considerations for marketing or distributing these programs to promote their uptake. To our knowledge, this is the first attempt to delineate these issues in this important target group for nicotine dependence intervention.

## Methods

### Design, Setting, and Review

We conducted a sequential exploratory, mixed methods study [19]. All research activities were conducted at the Group Health Research Institute (GHRI; currently known as the Kaiser Permanente Washington Health Research Institute) and approved by the Group Health Institutional Review Board. Qualitative interviews (phase 1) were conducted from April to May, 2016 to inform whether smokers who were not yet ready to quit were interested in using mHealth tools to modify their smoking behavior and, if so, get a preliminary sense of their desired program content, features, and marketing considerations. The results informed the design of a more comprehensive Web-based survey of smokers conducted from July to September, 2016 (phase 2). The Web-based survey was developed through an iterative process that included review by content experts and field testing with sample users to ensure face validity, user comprehension, and data integrity. All participants from both phases provided informed consent.

### Phase 1: Qualitative Interviews

#### Recruitment and Eligibility

Smokers (n=15) were recruited from the Greater Seattle area via Web-based Craigslist ads, community flyers, and from patients of Group Health Cooperative, a large, regional health care system in Washington state. Respondents were eligible if they (1) were 18 to 60 years old; (2) were current smokers interested in quitting someday, but not in the next month; (3) were able to speak and read in English; (4) had medical insurance; and (5) owned a smartphone which they used to access the Internet. Participants were recruited into 3 age categories: 18-29 years old (n=3), 30-39 years old (n=4), and 40 years or older (n=8). Each person participated in a phone interview and received US $50 as a thank you for their time.

#### Assessment, Coding, and Analysis

The interview guide was designed to elicit participants’ responses regarding their smoking and quit-attempt history, use of smartphones and mHealth apps, and ideal design and content for health-related mHealth apps including apps to help them cut back or quit smoking. Participants were also presented a list of 17 potential features and functions of an app designed to improve their health or help them stop smoking and asked to indicate which they be would be willing to use (yes or no) and why or why not. Items were modified based on a similar scale recently used with smokers and nicotine dependence clinicians [13]. Finally, participants were asked about how they would like to get or learn about mHealth apps (eg, from the app store, personal doctor, health plan or insurer, friends or family, or other).

Quantitative and demographic data were analyzed using descriptive statistics. Interviews were audio recorded, transcribed, and loaded in ATLAS.ti version 7 (ATLAS.ti Scientific Software Development GmbH) for coding and analysis. Qualitative feedback was analyzed using an inductive, conventional content analysis approach [20] and findings were interpreted in light of the three aims outlined previously (ie, interest in using apps, ideal features, and considerations for marketing or distribution).

#### Participant Characteristics

Participants were demographically diverse: 53% (n=8/15) were female, 27% (n=4/15) were Hispanic or Latino, 60% (n=9/15) were white, and 53% (n=8/15) had only a high school degree or less. Mean age was 37 years (range 19-54). Participants smoked an average of 12 cigarettes per day (range 4-20). Thirteen participants (87%) had previously tried to quit smoking, but none were interested in quitting in the next month. All participants owned and regularly used a smartphone.

#### Results

Participants overwhelmingly agreed that they would be interested in an mHealth app to assist them in both determining their readiness to quit smoking and providing assistance to help them successfully do so. Many noted that mHealth apps represent a “new approach,” unlike nicotine replacement therapy or quitting cold turkey, methods that several had tried in the past but were not successful. Some participants also noted that an mHealth app that helped them cut back, rather than quit smoking, was particularly appealing. As one participant (P4) stated, such an app, “ *would be great, especially to gradually reduce. (Gradually reducing) sounds so much easier than quitting...you ease into it.* ”

Based on the open-ended feedback and feature ratings, emergent themes were organized into three broad categories concerning participants’ recommendations for and perceptions of the utility of an mHealth app. They valued a program that would: (1) address smoking triggers; (2) build self-efficacy and accountability through social support and coaching; and (3) allow them to track their health behavior, set goals, and earn rewards. Key smoking triggers identified included stress, depression, anxiety, and the environment. One participant (P2) described her ideal program as one that would provide answers to questions like:

How would you create a safe environment to quit? How do you make your environment so you can quit? You know what I mean? Like what environment do you need so that you can quit? Like right now I’m talking to you and I didn't want the cigarette.

Several others suggested the app should provide ideas for alternative activities when they have cravings. For example, one (P7) suggested, “ *I would include tips...showing people or telling people other things to do, like craft or clean your house or go do your yard work to keep your mind off (smoking).* ” Another (P9) requested the ability to “ *contact somebody and get information on smoking, like what I can do or to help me with cravings*.”

In terms of building self-efficacy and accountability, participants described a fear of failure and belief that they did not possess the tools or ability to successfully quit or cut back on their smoking. In response, all but one said they would use an app that allowed them to talk with a health coach or counselor through private text messaging (short message service, SMS) or secure email built into the app. Two-thirds of participants were interested in receiving support from others. As one participant shared (P11), this type of outside support “ *might help me have more confidence to undertake something very difficult, you know, a little outside input that says, ‘You’re ready’ when I’m unsure on some level.* ”

Another person (P2) said they would like “ *networking with other people to quit...somewhere you can vent and whine. You never know, people might say something that might strike a chord with you like yeah, that’s so true*.”

But others were not interested in peer support or qualified their interest. For example, P7 commented, “ *If it were something like (a text message or email), I would be interested. But like say, joining a support group or even chatting online with other smokers? I don’t think I would do something like that*.” Another participant would only be interested in peer support if it did not come from family or friends. As this person (P13) explained, *“You don’t really want to show your friends you’re weak and need help, you want it to be more private.”*

Most participants were also interested in setting health goals, tracking their smoking and cigarette spending, or earning incentives for cutting back or quitting smoking. All of them said they would use a tool to track their smoking. This was seen as a way to help them cut back on their smoking, assess their readiness to quit, and monitor how much they smoke or spend over time.

An app to track how much I’m smoking would be great...like every time you smoke you tap a button and then over the course of a couple of weeks or even months it can tell you the ebb and flow

P11

...where it logs exactly, ‘Hey, you smoked this many cigarettes a day’ and then it turns it into a price...’You smoked $8 a day’ or whatever the case may be. And when you have something to look at and it expresses it to you, when somebody actually points out your fault, you're like, ‘Wow, I need to pay attention to that.’ It would let you know, ‘You know what? You over-smoked today. You’ve overspent.’

P1

Others suggested including features that allow users to track the health impacts of their smoking or quitting, *“...like ‘now that you've quit for this long, your lungs have regained this much’ so (it is) kind of like a motivator thing.”* [P15]

When asked how they would want to learn about an mHealth app for reducing smoking, most—but not all—participants identified their doctors as trusted informants. These participants described a scenario in which their doctors would introduce the app, but participants would download it themselves from the app store. One participant further described:

That would be so great if (the app recommendation) was on my going-home papers from (my health provider). If I saw (this), I would download it. There's always the quit smoking plan in the back of my after visit summaries, so if there was information about this app, that would be even more incentive than the number to call (to get help quitting).

P4

Participants who were reluctant to get the app from their doctor expressed concerns about cost. Another participant shared that she had not and was unwilling to tell her doctor she smoked, as she feared that her health care costs would go up. But there was a general willingness to download the app from an app store, either based on one’s own interest or at the suggestion of family or friends. One participant felt the app’s popularity could be spread through social networks:

Word of mouth is a great sales (tool) itself. If you really believe in your product or that app. My swamp game that I play, it's the dumbest game in the world, but...I got all my friends playing it just because of word of mouth.

P1

Additional participant preferences for each of the 17 features assessed are presented in Table 1. Of all of the potential features and topics assessed, smokers had the least interest in apps to help manage their diet or physical activity.

**Table 1:** Participant preferences for mHealth features.

Feature domain		Yes n (%)	No n (%)
**Addressing smoking triggers**			
	Advice for coping with cravings to smoke that is tailored specifically for you based on your needs or preferences	14 (93)	1 (7)
	Advice for handling stress that is tailored specifically for you based on your needs or preferences	13 (87)	2 (13)
**Building self-efficacy and accountability through social support and coaching**			
	The ability to talk with a health coach or counselor through private text messaging or secure email built into the app	14 (93)	1 (7)
	Information about the risks of smoking or benefits of quitting	12 (80)	3 (20)
	Information about stop-smoking medications, how they work, or how to get them	11 (73)	4 (27)
	Stories or videos from others talking about how they successfully changed their lifestyle and improved their health	11 (73)	4 (27)
	Social support from people other than your friends or family, like others who are trying to quit smoking or have successfully quit already	10 (67)	5 (33)
	Social support from people other than your friends or family, such as other people trying to change their diet or physical activity	9 (60)	6 (40)
	Social support from friends or family to help you stop smoking	9 (60)	6 (40)
	Social support from friend or family to help you change your diet or physical activity^a^	6 (40)	8 (53)
**Tracking health behavior, setting goals, and earning rewards**			
	A tool to track how many cigarettes you’ve smoked	15 (100)	0 (0)
	A tool to track how much money you spend on cigarettes or have saved by not smoking	13 (87)	2 (13)
	The ability to get points or credit for using the app and exchange them for rewards	13 (87)	2 (13)
	A tool to track your medication use	11 (73)	4 (27)
	A tool to track your physical activity	10 (67)	5 (33)
	A tool to track your diet	8 (53)	7 (47)
	Information about how to change your diet or physical activity^a^	7 (47)	7 (47)

^a^Totals do not add to 100% as 1 participant refused this question.

### Phase 2: Survey

#### Recruitment and Eligibility

Survey participants (n=116) were recruited via Craigslist ads and through ResearchMatch.org, a Web-based service funded by the US National Institutes of Health Clinical and Translational Science Award (CTSA) program which matches prescreened volunteers with relevant medical research studies. Advertisements were placed in 21 states representing all US geographic regions, but focused more heavily on states in the southeast and midwest due to higher smoking prevalence in these regions [21]. Persons interested in learning more about the study were directed to a study website where they were screened for eligibility, provided consent, and completed the survey. Completely Automated Public Turing test to tell Computers and Humans Apart (CAPTCHA) verification was used to exclude nonhuman respondents. Persons were eligible if they (1) were 18-60 years old; (2) were current smokers interested in quitting someday, but not in the next month; (3) were able to speak and read in English; (4) owned a smartphone; and (5) used any app on their phone. Participation was limited to 1 survey per person, enforced with a combination of cookies and cross-referencing participant names and email addresses to ensure that there were no duplicates. A total of 250 people were screened for eligibility; of which, 123 screened ineligible (primarily because they were trying to quit smoking or had plans to quit within the month; n=92), and 1 person broke off before completing screening. Additionally, 10 eligible respondents declined participation, leaving 116 enrolled participants. Participants were provided a US $20 Amazon gift card code as a thank you for their time.

#### Assessment Measures

Participants were asked about their demographics, smoking and quit attempt history, use of smartphones and mHealth apps, interest in smoking-focused mHealth apps, and reasons they were or were not interested in these tools. Participants were presented a list of 42 specific mobile app features, functionality, and content topics and asked to rate how important or how appealing they found each of them. Item selection was based on a similar scale previously used to assess interest in mHealth app content and features among smoking cessation treatment experts and smokers [13,14], but modified to include additional response options based on the target audience, phase 1 results, and input from the authors. Importance and appeal were each rated on a 4-point Likert scale ranging from “not at all” to “very” important or appealing. Participants could also write in additional desired features, functions, or content.

#### Analysis

Survey responses were analyzed using SPSS version 22 (IBM Corporation). Descriptive statistics were used to summarize overall results. Preferences for mHealth features, functions, and content were also compared by stage of change (precontemplators vs contemplators) using Pearson chi-square analyses given the categorical nature of the ratings. Multiple comparisons were adjusted using the Bonferroni correction. Write-in comments were coded for common themes and summarized.

### Phase 2 Results

#### Participants

##### Demographics and Smoking

Participant demographics and smoking characteristics are presented in Table 2. Most were moderate smokers, middle-aged, female, white, had a college degree, and a household income under US $50,000 a year. Participants were recruited from a total of 29 states and Washington, the District of Columbia. States represented all geographic regions of the continental United States. Few used tobacco other than cigarettes. Forty participants (34.5%, 40/116) were recruited via Craigslist and 76 (65.5%, 76/116) via ResearchMatch.org.

##### Interest in Reducing or Quitting Smoking

One-third (37.1%, 43/116) had attempted to quit smoking in the past year. Whereas participants were not actively attempting or planning to quit smoking in the near term, most agreed that they would cut back on their smoking if they knew where to find help (59.4%, 69/116) and nearly half said they would quit smoking if they knew where to find help (47.4%, 55/116).

##### Mobile Phone Use

Participants were predominantly Android users (75.0%, 87/116); only 29 (25.0%, 29/116) owned an iPhone. The majority actively used their mobile phones for accessing the Internet (97.4%, 113/116), taking pictures (97.4%, 113/116), sending email (95.7%, 111/116) and text messages (93.1%, 108/116), downloading apps (92.2%, 107/116), playing games (86.2%, 100/116), and listening to music (84.5%, 98/116).

**Table 2:** Participant characteristics.

Characteristics		Participants (N=116)
**Gender, n (%)**		
	Female	84 (72.4)
**Race and Ethnicity, n (%)**		
	Hispanic or Latino	8 (6.9)
	White	80 (69.0)
	Black	19 (16.4)
	Asian	5 (4.3)
	American Indian or Alaska Native	2 (1.7)
	Other	5 (4.3)
	Decline	5 (4.3)
**Education, n (%)**		
	High school degree or general educational development (GED) certificate	48 (41.4)
	College degree	58 (50.0)
	Graduate degree	8 (6.9)
	Household income < US $50,000	65 (56.1)
**Other regular nicotine or tobacco use, n (%)**		
	Electronic cigarettes	16 (13.8)
	Cigars or cigarillos	9 (7.8)
	Hookah	14 (12.1)
	Quit attempt in past year (yes)	43 (37.1)
**Stage of change, n (%)**		
	Precontemplation	37 (32.2)
	Contemplation	78 (67.8)
Age, mean (SD)		38.1 (11.7)
Cigarettes per day, mean (SD)		15.5 (12.9)

#### Interest in mHealth Apps

Approximately half (55.2%, 64/116) had used an app to manage 1 or more common health-related issues. Nearly half (45.7%, 53/116) had used a physical activity app. Twenty-five people (21.6%, 25/116) had used an app to track their food, calories, or weight. Eight people (6.9%, 8/116) had used a stress reduction app, 11 (9.5%, 11/116) used an app to track their sleep, 9 (7.8%, 9/116) used an app to help them manage their mood, and 3 people (1.2%, 3/116) had used another health-related app. However, only 4 (3.4%, 4/116) had ever downloaded an app to help them stop smoking.

In contrast, most (75.0%, 87/116) said they would consider downloading an app to help them stop smoking. Among those who said they would not consider this or were unsure whether they would ever consider this, many (44%, 11/25) expressed uncertainty that an app could help them change their behavior. Comments included, “*Other health apps I have used have not been particularly helpful*,” “ *I’m unsure how an app would aid in the prevention of smoking*,” and “*I don’t consider technology to be a good source to make someone do something*.” Less frequent concerns expressed included cost, a reticence to download apps in general, and a lack of interest in quitting smoking.

More people (87.9%, 102/116) were interested in an app that could help them reduce their smoking than stop smoking (75%, 87/116). This interest was driven by a desire to improve one’s health 71.6%, 83/116), protect one’s future health (62.9%, 73/116), and save money (61.2%, 71/116). Similarly, nearly all participants expressed interest in an app that could help them decide “if, when, or how” to quit smoking (90.5%, 105/116). Of these 3 topics, more people were interested in knowing how to quit (51.7%, 60/116) than getting help deciding if they were interested in quitting (11.2%, 13/116) or when would be a good time to quit (26.7%, 31/116). People who already owned health-related apps were more willing to download an app to help them decide if, when, or how to quit smoking than those who did not own health apps (51.8%, 61/116 vs 41.9%, 44/116; *X*^2^_4_ [n=116]=22.0, *P*<.001). A similar relation was observed for willingness to download an app to help people reduce their smoking. More people who already owned health-related apps were interested in this than people who did not own health apps (57.8%, 59/116 vs 41.9%, 44/116; *X*^2^_4_ [n=114]=12.2, *P*=.02). Willingness to use a smoking reduction app did not vary by education, income, race, or age. Interest in an app to help people decide if, when, or how to quit smoking varied by income, but not by education, age, or race. Most of the participants who were interested in this type of app reported an annual household income between US $25,000 to US $50,000 (30.8%, 32/116; *X*^2^_10_ [n=115]=18.4, *P*=.05).

#### Preferred Features, Functionality, and Content of mHealth Smoking Apps

##### Privacy and Security Features

Participants rated the importance of various privacy and security features they would want to see in an app to help them change their smoking behavior (Table 3). They did not express a clear preference for blocking access to personal information stored in other apps versus allowing the app to access this information with permission; both were rated as important features. Similarly, they did not express a clear preference for whether their program information should be stored locally on their phone or saved in “the cloud” to allow access from other devices; both of these were seen as less important than other privacy and security considerations. Overall, password protection was viewed as important, with 41% of participants rating this as “very important.” Preferences for these features did not vary significantly by stage of change.

**Table 3:** Perceived importance of privacy and security features. Items are measured on 4-point Likert scale from “not at all” to “very” important.

Feature	Mean (SD)	Rated “very important” n (%)	Rated “not at all important” n (%)
App does not access personal information on phone (eg, contacts, calendar, Facebook)	3.09 (0.97)	51 (44)	8 (6.9)
App can access personal information on phone, but I can decide which	3.07 (0.95)	46 (39.7)	10 (8.6)
App is password protected	2.99 (1.03)	48 (41.4)	12 (10.3)
My data is stored in “the cloud” so I can access from other devices	2.62 (1.05)	30 (25.9)	20 (17.2)
My data is stored on phone and not in “the cloud”	2.38 (1.07)	22 (19.0)	30 (25.9)

##### Functionality

Participants also rated the relative appeal of a range of potential app functionality (Table 4). Tracking functions were viewed as relatively important overall, with a 38% to 54% of participants rating these functions as “very appealing.” Participants preferred content that could dynamically update to match their changing needs and interests, were interested in receiving rewards, and liked the idea of being able to request support or advice through the program when they needed it or to get immediate advice after answering a brief survey, but other forms of support and connectivity were rated as less appealing overall. In particular, smokers rated the ability to share updates with family or friends via social media or to video chat with other smokers or treatment experts as least appealing; 34% to 47% of participants rated these features as “not at all” appealing.

Participants were asked what type of rewards they would want to receive in exchange for points accumulated from viewing program content or completing tasks. Most (87.0%, 100/115) preferred a gift card or money, 9 people (7.8%, 9/115) wanted nicotine replacement patches, 3 (2.6%, 3/115) preferred free advice from a stop-smoking counselor or doctor, 2 (1.7%, 2/115) were interested in receiving another app of their choosing, and 1 person (0.9%, 1/115) simply wrote “gold.”

There was a significant relationship between stage of change and the appeal of reporting one’s progress on social media (*X*^2^_3_ [n=114]=13.1, *P*<.01). More precontemplators rated the ability to share their progress on social media as “not at all” or only “somewhat” appealing (83.7%, 31/116) compared with 66.2% (51/116) of contemplators. Other item comparisons by stage of change were not significantly different.

**Table 4:** Perceived appeal of potential app functions. Items were measured on 4-point Likert scale from “not at all” to “very” appealing.

Function domain			Mean (SD)	Rated “very appealing” n (%)	Rated “not at all appealing” n (%)
**Tracking**				
	Tracks how much I save by not smoking		3.34 (0.83)	63 (54.3)	2 (1.7)
	Tracks how much I spend on smoking		3.26 (0.88)	57 (49.1)	6 (5.2)
	Tracks how much I smoke		3.06 (0.92)	44 (37.9)	8 (6.9)
**Static versus dynamic**				
	Content adapts over time to my needs or interests		3.17 (0.75)	40 (34.5)	3 (2.6)
	Content stays the same and does not change		2.14 (0.89)	8 (6.9)	30 (25.9)
**Support and connectivity**				
	Lets me request support or advice when I need or want it		3.16 (0.84)	48 (41.1)	3 (2.6)
	Can get immediate advice after answering a brief survey		2.92 (0.90)	34 (29.3)	8 (6.9)
	Includes advice from stop-smoking experts		2.88 (0.89)	31 (26.7)	8 (6.9)
	Includes stories from other smokers with support and advice		2.63 (1.01)	27 (23.3)	18 (15.5)
	Sends me motivational or supportive messages via text message		2.63 (0.99)	24 (20.7)	18 (15.5)
	Let me text or email other smokers for support and advice		2.60 (1.03)	27 (23.3)	20 (17.2)
	Can request advice, but may wait 24-48 hours for response		2.43 (1.03)	20 (17.2)	26 (22.4)
	Sends me motivational or supportive messages via email		2.42 (1.00)	20 (17.2)	23 (19.8)
	Lets me send private messages to my doctor		2.39 (0.97)	18 (15.5)	22 (19.0)
	Lets me share my progress with family and friends		2.18 (1.00)	13 (11.2)	35 (30.2)
	Lets me video chat with stop-smoking experts		2.14 (1.05)	17 (14.7)	39 (33.6)
	Lets me video chat with other smokers		2.03 (1.04)	13 (11.2)	47 (40.5)
	Lets me share my progress on Facebook, Twitter, or social media^a^		1.90 (1.01)	10 (8.6)	55 (47.4)
**Rewards**				
	Lets me earn points to redeem for free gifts		3.45 (0.83)	74 (63.8)	3 (2.6)
	Lets me earn points or badges to track progress		3.11 (0.94)	50 (43.1)	8 (6.9)

^a^Significant difference by stage of change.

##### Content and Focus

Participants rated the appeal and perceived importance of different content which might be included in an app to help them either reduce their smoking or decide if, when, or how to stop smoking (Table 5). Nearly half (47%) said that an app to help them decide how to stop smoking was “very appealing” and more than half (53%) rated help managing nicotine withdrawal as “very appealing.” A substantial portion of participants (ranging from 39%-53%) also thought it was very important that a smoking-focused app should also include information about related issues like stress reduction, help managing depression and anxiety, or help managing their weight. However, the appeal of stress management content varied by stage of change (*X*^2^_2_ [n=114]=7.9, *P*=.02). This feature was viewed as “very important” by 70.3% (26/116) of precontemplators versus 44.2% (34/116) of contemplators. Whereas not statistically significant (*X*^2^_3_ [n=114]=7.0, *P*=.07), more precontemplators rated help managing stop-smoking medication side-effects as appealing or very appealing (72.9%, 27/116 vs 57.2%, 44/116).

Games were considered relatively important among everyone (mean=2.85 out of 4), but overall, fewer people considered these “very important” than considered health related content as “very important” (Table 5).

Participants were asked what other features they would like to see in an app designed to help them cut back or quit smoking. Twenty three stated they had no additional suggestions. Seventy-seven participants provided written suggestions. Among these, the most common theme (n=15) was the ability to track one’s behavior (cigarettes smoked, purchased), health status (improvements over time), or money (amount spent on cigarettes or saved by not smoking). The second most common themes, each endorsed by 5 people, was the ability to earn rewards by using the program or to somehow distract themselves from smoking. Four respondents requested a place to journal about their experience or record their own positive affirmations and 4 people wanted some type of interaction with other smokers. Suggestions for the latter included stories from other smokers, the ability to get advice from others, and the ability to track others’ milestones without having to personally interact with them. The remaining responses were only endorsed once each and included offering standard treatment content such as information on the risks and benefits of smoking, as well as more controversial suggestions such as providing “electric shock” and including images of smokers’ diseased lungs.

**Table 5:** Perceived appeal and importance of content. Items were measured on 4-point Likert scale from “not at all” to “very.”

Content domain		Mean (SD)	Rated “very appealing” n (%)	Rated “not at all appealing” n (%)
**Focus**				
	Guides me “how” to quit	3.26 (0.84)	55 (47.4)	4 (3.4)
	Helps me cut-back but not quit	2.95 (0.86)	34 (29.3)	5 (4.3)
	Helps me decide “if” I want to quit	2.57 (0.97)	21 (18.1)	18 (15.5)
**Stop-smoking content**				
	Helps me manage nicotine withdrawal	3.35 (0.78)	61 (52.6)	1 (0.9)
	Helps me manage medication side-effects	2.78 (0.95)	30 (25.9)	12 (10.3)
	Includes information on stop-smoking medications	2.63 (0.87)	19 (16.4)	11 (9.5)
**Nonsmoking content**				
	Helps manage stress^a^	3.40 (0.74)	61 (52.6)	18 (15.5)
	Helps manage anxiety	3.30 (0.85)	59 (50.9)	3 (3.4)
	Helps manage depression	3.17 (0.85)	47 (40.5)	5 (4.3)
	Helps manage weight	2.97 (1.01)	45 (38.8)	12 (10.3)
	Games for fun or distraction from smoking	2.85 (0.93)	34 (29.3)	8 (6.9)

^a^Significant difference by stage of change.

#### Considerations for Marketing and Distribution

##### Source and Reputation

Participants rated the importance of different reputational factors that might influence their decision to use a smoking-related app (Table 6). Based on the mean Likert scale score, most of the reputational metrics assessed were deemed “important,” but fewer people rated a recommendation from their personal doctor as “very important” than the other considerations assessed. The most important consideration, rated as “very important” by 48% of people, was that the app be research-tested. Opinions on source and reputation did not vary by stage of change.

**Table 6:** Perceived importance of reputational metrics. Items were measured on 4-point Likert scale from “not at all” to “very” important.

Metrics	Mean (SD)	Rated “very important” n (%)	Rated “not at all important” n (%)
Research tested	3.22 (0.88)	56 (48.3)	4 (3.4)
Recommended by treatment experts	3.16 (0.88)	50 (43.1)	5 (4.3)
Highly rated by others	3.06 (0.95)	50 (43.1)	6 (5.2)
Developed by a trusted source	3.04 (0.95)	47 (40.5)	7 (6.0)
Recommended by my doctor	2.70 (1.03)	34 (29.3)	15 (12.9)

##### Price

Most smokers were not willing to pay much for an app to help them cut back on their smoking or decide if, when, or how to quit (Table 7). One third said they would not be willing to pay anything. Fewer than 4% would pay more than US $10 for this intervention program. No difference was observed by stage of change.

**Table 7:** Maximum price willing to pay for smoking-related mHealth apps.

Maximum willing to pay	App to reduce smoking n (%)	App to decide if, when, or how to quit n (%)
US $0	46 (39.7)	39 (33.6)
US $1	13 (11.2)	14 (12.1)
US $2	23 (19.8)	21 (18.1)
US $5	23 (19.8)	24 (20.7)
US $10	7 (6.0)	13 (11.2)
US > $10	4 (3.4)	5 (4.3)

## Discussion

To our knowledge, this is the first report to assess whether smokers who are not yet ready to quit are interested in using mHealth apps to change their smoking behavior or inform their decisions about smoking. Others have examined technology use among smokers who are not motivated to quit, but did not look at use of smoking cessation apps specifically [22]. Similarly, increasing attention is being focused on how to design appealing and effective mHealth programs for smokers [13-18] but this work has focused on smokers who are ready to quit. As such, this paper makes a unique contribution to the literature.

### Principal Findings

All phase 2 survey participants were smartphone owners and most regularly used apps; however, relatively few had used common mHealth apps and only 3% (4/116) had ever downloaded a cessation app. This supports our contention that smokers who are not yet ready to quit are not likely to proactively download and use traditional cessation-focused smoking apps. Similar results were found in a recent survey of US smokers; only 6% of smartphone owners who smoked and were not motivated to quit had used a cessation app versus 24% of those who were motivated to quit [22].

As in our prior research with smokers who are not yet ready to quit [10-12], both phase 1 and phase 2 participants were interested in getting assistance in changing their smoking behavior, even though they were not ready to commit to quitting. Notably, 88% of phase 2 survey respondents expressed interest in an app to help them reduce their smoking and 91% expressed interest in an app to help them decide “if, when, or how” to quit, with nearly half of participants saying that learning how to quit was “very appealing.” This suggests that mHealth tools targeting this population should have a broader focus than cessation, even though the content should still help users understand the process of quitting for when they are ready. It is equally notable that almost a third of participants wanted a tool to simply help them cut back on their current smoking and nearly 1 in 5 would like a tool to help them decide “if” they want to quit. Thus, the optimal program for this population needs to address a range of user goals.

Participants rated their preferences for a variety of potential mHealth features, functions, and content. We suggest that items with a mean score of 3 out of 4 (indicating an average rating of “important or appealing” or “very important or appealing”) reflect items most highly valued. With little exception, these items were also rated as “very” important or appealing by at least 40% of respondents. Using this metric, the app features, functions, and content rated most highly were: (1) security of personal information (eg, password protection and no or limited access to personal information on one’s phone); (2) the ability to track smoking, spending, and savings; (3) content that adaptively changes with one’s needs and preferences; (4) the ability to request support as needed; (5) the ability to earn and redeem awards for program use; (6) guidance on how to quit smoking; and (7) content specifically addressing management of nicotine withdrawal, stress, depression, and anxiety. Many of these themes emerged during the phase 1 interviews as well. With the exception of the security features and incentives, these are standard components of cognitive behavioral nicotine dependence programs [23]. In that respect, smokers not yet ready to quit are interested in much of the same information known to be important with smokers who are ready to quit but the way this information is framed may need to differ to appeal to smokers who are still unsure of their quitting goals. For example, 1 phase 2 participant commented, “ *I like that you are asking ‘if’ I want to quit rather than stopping right away*.” This illustrates the importance of respecting that not everyone is ready to quit or necessarily wants to stop smoking, even if they do want to cut back or change their smoking behavior. Thus, apps designed for this target group should acknowledge that ambivalence.

Participants’ interest in tracking tools was a consistent theme in phases 1 and 2 and was echoed in the phase 2 write-in comments. In fact, tracking was the most common write-in theme endorsed. In addition to tracking the typical financial and smoking metrics, participants suggested they would like to track health changes over time. A similar theme emerged during the phase 1 interviews. As wearable sensors become more advanced and available, we could envision a future system that might track users’ heart rate, pulse, or oxygen saturation as relevant indices of health improvement when one cuts back or quits smoking. Our phase 1 participants indicated this type of feedback would be motivating, although it is worth pointing out that using biologically-based metrics of harm exposure or risk to motivate behavior change, including smoking cessation, has yielded mixed results when empirically studied [24-28]. Thus, these features may sound appealing but the potential for their actual impact on cessation is unclear. However, remote sensors could be used to monitor smoking events and event geolocation of these events. In turn, this information could be used to help smokers better understand when, where, and perhaps why they smoke. This insight may be useful in quitting.

Perhaps equally important to participants’ preferred features and functions was their lack of interest in sharing their progress via social media; nearly half rated this as “not at all” appealing and it received the lowest overall mean score (1.9 out of 4). This finding echoes the opinion of smokers in another recent survey [13] and reflects the sentiment shared by one of the phase 1 participants that, “ *You don't really want to show your friends you're weak and need help, you want it to be more private.”* In contrast, many recent studies are testing social media platforms to intervene with smokers [29-33]. These programs may ultimately be most appealing when sharing is limited to a closed group of users and not shared broadly with one’s friends and family. It is also possible that social media will have greater appeal among younger smokers than older smokers, since younger people may be more comfortable sharing personal information over social media, in general. But it remains unclear if even younger smokers who have not yet committed to quit smoking will be interested in sharing their experiences in this way.

Finally, we sought to better understand future marketing or distribution considerations. This will perhaps be the greatest challenge of intervening with precontemplative and contemplative smokers, as it is unclear if smokers who are not yet ready to quit will voluntarily seek out tools to help them decide if or how to quit, even though they expressed interest in these tools in our survey. Whereas our findings do not fully inform how to connect smokers with these tools, it is clear that cost will be a barrier. Sixty percent of respondents said they would be willing to pay but our data suggest the cost should be under US $5. This is consistent with a general trend of price sensitivity for mobile apps. Program source, expert recommendations, empirical validation, and user ratings also appear important considerations for smokers and should be highlighted in promotional materials. Opinions about the role of health care providers in distributing these materials differed between the phase 1 and 2 participants but doctors, health care systems, and insurers could play a role in educating patients about the availability of a relevant mHealth app, even if not directly distributing it.

### Strengths and Limitations

This work has a number of strengths including the use of a sequential exploratory mixed-methods analysis, nationwide recruitment, inclusion of smokers who are active smartphone users, and its focus on smokers who are not yet ready to quit. The latter makes up the majority of smokers, yet little is known about how best to engage these individuals in treatment or their preferences for using mHealth tools.

The chief limitation of this study is the small sample size, which limits generalizability. Compared with all US smokers assessed via the 2012-2014 National Health Interview Survey (NHIS) [34], our sample included more females (72.4% vs 45.3%) but fewer Hispanics (6.9% vs 10.3%) and whites (69.0% vs 81.0%). Slightly more than half (55.9%) of the people in our sample had a college degree or higher education compared with slightly less than half (44.7%) of those in the NHIS sample who had some or more college education. The groups also differed in terms of age (for those age groups included in our sample, we excluded people over 60 years). Among all US smokers, 12.8% are aged 18-25 years versus 11.2% of our sample, 45.3% are aged 25-44 years versus 56.0% of our sample, and 40.1% are aged 45-64 years compared with 32.8% of our sample. However, the groups are comparable in terms of the percentage of persons who smoke 20 or more cigarettes a day (28.9% NHIS vs 28.5% in our sample). These differences are not surprising since our sample was limited to adult smokers who regularly use smartphones and were not yet ready to quit smoking. Since nationally-representative data are not available on this specific subgroup of smokers, we cannot say to what extent the findings will generalize to this population of interest. However, the consistency of themes observed across phases 1 and 2 lend credence to the general validity of the results among similar US smokers.

Finally, we note that the preferences expressed by smokers in this study are based on the features and functionality they expect they would like. User preferences could be different if assessed in reaction to an actual app reflecting these preferred features, particularly if reactions are assessed based on “real-world” user conditions. But the results of this study provide some initial guideposts for developing these tools in the future.

### Conclusions

This mixed-methods study confirmed that smokers who are not yet ready to quit are receptive to using mHealth tools to reduce or quit smoking. As such, these smokers represent an important target group for mHealth delivered interventions. In addition to being designed with an understanding of best practice nicotine dependence treatment, mHealth apps should be designed to appeal to smokers who have not yet committed to quitting. Findings from this study can provide insight into how to achieve this goal and may aid addiction treatment experts, design specialists, and software developers interested in creating new public-health focused smoking cessation apps in the future.

## Abbreviations

mHealth: mobile Health
